# Natural History of Perceived Food Hypersensitivity and IgE Sensitisation to Food Allergens in a Cohort of Adults

**DOI:** 10.1371/journal.pone.0085333

**Published:** 2014-01-10

**Authors:** Antonios Patelis, Maria Gunnbjörnsdottir, Magnus P. Borres, Peter Burney, Thorarinn Gislason, Kjell Torén, Bertil Forsberg, Kjell Alving, Andrei Malinovschi, Christer Janson

**Affiliations:** 1 Department of Medical Sciences: Respiratory Medicine and Allergology, Uppsala University, Uppsala, Sweden; 2 Immunodiagnostics, Thermo Fischer Scientific, Uppsala, Sweden; 3 University of Iceland, Medical Faculty and Department of Respiratory Medicine and Sleep, Landspitali University Hospital, Reykjavik, Iceland; 4 Section of Occupational and Environmental Medicine, Institute of Medicine, University of Gothenburg, Gothenburg, Sweden; 5 Division of Occupational and Environmental Medicine, Umeå University, Umeå, Sweden; 6 Department of Women's and Children's Health, Uppsala University, Uppsala, Sweden; 7 Respiratory Epidemiology and Public Health, Imperial College, London, United Kingdom; 8 Department of Medical Sciences: Clinical Physiology, Uppsala University, Uppsala, Sweden; Mario Negri Institute for Pharmacological Research and Azienda Ospedaliera Ospedali Riuniti di Bergamo, Italy

## Abstract

**Background:**

No longitudinal studies exist on the natural history of food hypersensitivity and IgE sensitisation to food allergens in adults.

**Objective:**

To examine the natural history of food hypersensitivity, the natural history of IgE sensitisation to food allergens and to investigate the risk factors for new onset food hypersensitivity.

**Methods:**

Food hypersensitivity was questionnaire-assessed in 2307 individuals (aged 20–45 years) from Iceland and Sweden during the European Community Respiratory Health Survey both at baseline and follow-up 9 years later. IgE food and aeroallergen sensitisation were assessed in a subgroup of these individuals (n = 807). Values of 0.35 kU/L and above were regarded as positive sensitisation.

**Results:**

Food hypersensitivity was reported by 21% of the subjects and this proportion remained unchanged at follow-up (p = 0.58). Fruits, nuts and vegetables were the three most common causes of food hypersensitivity, with a similar prevalence at baseline and follow-up. The prevalence IgE sensitisation to food allergens decreased in general by 56% (p<0.001) and IgE sensitisation to peanut decreased in particular by 67% (p = 0.003). The prevalence of timothy grass IgE sensitisation decreased by 15% (p = 0.003) while cat, mite and birch IgE sensitisation did not decrease significantly. Female sex, rhinitis, eczema and presence of IgE sensitisation to aeroallergens were independently associated with new onset food hypersensitivity.

**Conclusion:**

The prevalence of food hypersensitivity remained unchanged while the prevalence of IgE sensitisation to food allergens decreased in adults over a 9-year follow-up period. The decrease in prevalence of IgE sensitisation to food allergens was considerably larger than the change in prevalence of IgE sensitisation to aeroallergens.

## Introduction

Several population surveys in the 1990s have estimated the prevalence of perceived food hypersensitivity, e.g. self-reported adverse reactions after food ingestion, regardless of the underlying mechanism [1], at 12% to 20% in adults [2]–[4]. The majority of adverse reactions to food are not IgE-mediated [5] and there is limited information on the prevalence and distribution of IgE food sensitisation. About 15% to 20% of the population has IgE to at least one food allergen over the detection limit [6], [7] e.g. food IgE sensitisation. However, only a small fraction of subjects with IgE antibodies against food allergens have clinical IgE-mediated food allergy. As an example, Liu et al [7] reported that the prevalence of food IgE sensitisation was 15% whereas the prevalence of IgE-mediated food allergy was only 2%.

Aeroallergen IgE sensitisation, especially against perennial allergens, is linked to asthma and airway inflammation [8]–[11]. The natural history of prevalence of aeroallergen IgE sensitisation has been examined in longitudinal studies in adults [12]–[18], but the results are contradictory and show the prevalence decreasing [15], [18], remaining stable [12], [19] or increasing [13], [14], [16], [17] over time.

To the best of our knowledge, no study exists about the natural history of food hypersensitivity and IgE sensitisation to food allergens in adults. Another reason for studying IgE sensitisation to food allergens and IgE-mediated food allergy is that not only aeroallergen sensitisation, but also food IgE sensitisation, is linked to increased exhaled nitric oxide levels and increased risk for asthma [6], [7], [20].

The aim of this study was to examine the natural history food hypersensitivity symptoms and IgE sensitisation to food allergens and to investigate the risk factors for new onset food hypersensitivity and sensitisation.

## Methods

### Population

**Figure 1:**
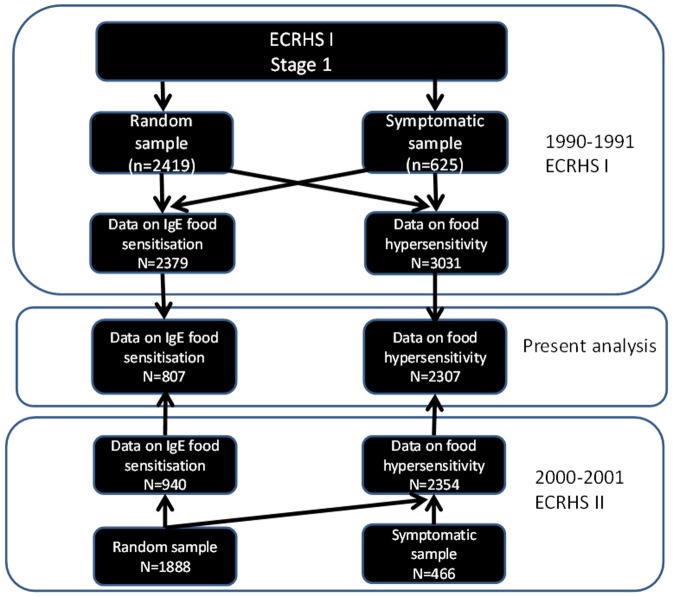
Flow chart explaining the selection of the studied population.

The study was based on the European Community Respiratory Health Survey (ECRHS) I (1991–92) and II (2000–01) [21] from four participating centres (Reykjavík, Uppsala, Gothenburg and Umeå). Each participant in ECRHS I was sent a brief questionnaire (Stage 1) and among those who responded, both a random and a ‘‘symptomatic’’ sample (consisting of subjects with symptoms of asthma or asthma medication), were invited to undergo a more detailed clinical examination (Stage 2). Subjects from Stage 2 of ECRHS I were invited to participate in the follow-up study, ECRHS II, and answered a standardized questionnaire and underwent a clinical visit [21]. A total of 2,307 individuals answered questions about food hypersensitivity symptoms both at ECRHS I and II. No difference in age, sex, BMI, prevalence of asthma, rhinitis, total IgE, food IgE sensitisation, aeroallergen IgE sensitisation and smoking history were found between subjects with and without follow-up data about food hypersensitivity symptoms (all p>0.05). A subgroup of ECRHS participants in Iceland (Reykjavík) and Sweden (Uppsala, Gothenburg and Umeå) from the random sample (n = 807) was examined against IgE to food allergens at ECRHS I [22] [23] and ECRHS II (Figure 1).

### Questionnaires

The main questionnaire used in ECRHS I and II http://www.ecrhs.org [24] gathered information about respiratory symptoms, exposure to investigated allergens, and smoking history.

### Diagnosis of asthma

A person was defined as asthmatic if he/she had ever been diagnosed with asthma and had an asthma attack or one of the following symptoms: nocturnal chest tightness, attack of shortness of breath, chest wheezing or whistling during the last 12 months [25].

### Definition of food hypersensitivity

An individual was considered having food hypersensitivity [1] if he/she answered yes to the following questions “Have you ever had an illness or trouble caused by eating a particular food or foods?” and if so, “Have you nearly always had the same illness or trouble after eating this type of food?” Respondents were then asked to list the food(s) and symptoms [2].

### Measurements of IgE to aeroallergens and food allergens and total IgE

Venous blood samples were drawn from all participants in the clinical visits and frozen at −20°C for measurement of total serum IgE (total IgE) and serum IgE antibodies to cat, *D. pteronyssinus*, timothy grass, birch, and *Cladosporium*, as part of the ECRHS I and II protocol [21]. Measurement of IgE antibodies against birch was performed in all centres in the ECRHS I and in the Uppsala centre in ECRHS II. In addition, IgE antibodies to a mix of food allergens (containing egg white, milk, cod fish, wheat, peanut, and soybean) were measured in the subgroup of ECRHS participants in Iceland (Reykjavík) and Sweden (Uppsala, Gothenburg and Umeå) from the random sample by same technique and reagents at ECRHS I and II (Pharmacia CAP System, MultiCap fx5, Thermo Fisher Scientific, Uppsala, Sweden). Serum with a positive reaction to the food panel (fx5) was further analysed for the single allergens in the panel. The results are presented in kU/L, and values of 0.35 kU/L and above were regarded as positive sensitisation [26]. A titre below the detection level (<0.35 kU/L) was arbitrarily given the value 0.17 kU/L). All samples from ECRHS I from Reykjavík, Uppsala, Gothenburg and Umeå were analysed in Uppsala, as previously described [22] while samples from the random sample of ECRHS II (depending on availability of residual samples) were analysed within the frame of EuroPREVALL in Manchester, as described elsewhere [27].

### Statistics

Statistical analyses were performed using STATA 12.1 software (Stata Corp., 2011, Texas, USA). IgE titres (both total and specific) were log transformed before analysis. Mc Nemar's test was used to compare prevalence of food hypersensitivity, various food related symptoms and IgE sensitisation in ECRHS I and II. Chi-squared test (for categorical variables) and unpaired t-test (for numerical variables) were used to compare individuals with new onset and no food hypersensitivity and prevalence of IgE sensitisation to food, cat/mite and timothy grass allergens. Chi-squared test was also used to compare prevalence of IgE sensitisation to food in subjects according to age (< or > median age). Paired t-test was used to compare levels of IgE to specific allergens between ECRHS I and II in subjects sensitised to each respective allergen at either ECRHS I or II and unpaired t-test was used to compare levels of IgE against food allergens at ECRHS I in subjects with persistence and remission of IgE sensitisation to food allergens. Multiple logistic regression models were used when examining predictors for new onset food hypersensitivity, and persistent food sensitisation. These models always included age, gender, body mass index, rhinitis, current asthma, total IgE, IgE against food allergens, sample and IgE against aeroallergens. A p–value of <0.05 was considered statistically significant.

### Ethics

All subjects consented to the utilization of personal data for the purpose of this study. The study was approved by the Ethics Committee at the Medical Faculty at Uppsala University and University of Iceland.

## Results

The study population comprised of 2,307 individuals who answered questions about food hypersensitivity symptoms in both ECRHS I and II. Of these, 53% were women. The group characteristics of the participants are presented in Table 1.

**Table 1:** Characteristics of the participants (n = 2,307; mean ± SD and n (%)).

	ECRHS I 1991–92	ECRHS II 2000–01	p value
Age	33.6 ±7.3	42.4 ±7.2	<0.001
BMI	23.8 ±3.6	25.7 ±4.2	<0.001
Current asthma	314 (13.4)	428 (18.3)	<0.001
Rhinitis	783 (33.3)	904 (38.5)	<0.001
Smoking history			<0.001
Never	1093 (46.6)	1044 (45.9)	
Ex	54 (23.1)	730 (32.2)	
Current	711 (30.3)	497 (21.9)	

### Natural history of food hypersensitivity symptoms

Around 21% of the interviewed subjects reported food hypersensitivity symptoms in both ECRHS I and II (Table 2). The most common foods related to these symptoms were fruits followed by nuts and then vegetables. The prevalence of food hypersensitivity in relation to ingestion of fish (including seafood and shellfish) increased significantly from ECRHS I to II and become, together with vegetables, the fourth most common cause of food hypersensitivity. Food hypersensitivity symptoms due to dairy products, wheat products (gluten, cereal and wheat) and spices (herbs, chili, and garlic) also increased significantly from ECRHS I to II (Table 2).

**Table 2:** Prevalence (n (%)) of type of food reported as cause of food hypersensitivity (n = 2,307).

	ECRHS I 1991–92	ECRHS II 2000–01	p value
Any food	496 (21.5)	508 (22.0)	0.58
Fruits	195 (8.5)	215 (9.3)	0.19
Nuts	126 (5.5)	135 (5.9)	0.47
Vegetables	78 (3.4)	97 (4.2)	0.11
Fish, seafood, shellfish	56 (2.4)	97 (4.2)	<0.001
Chocolate	27 (1.2)	23 (1.0)	0.65
Egg	24 (1.0)	19 (0.8)	0.34
Milk and dairy products	22 (1.0)	57 (2.5)	<0.001
Meat	11 (0.4)	19 (0.8)	0.14
Herbs, chilli, garlic	9 (0.3)	20 (0.9)	0.004
Gluten, cereal, wheat products	5 (0.3)	14 (0.6)	0.049

Skin reactions were the most common reported symptoms both at baseline and follow-up (Table 3). No significant change in the prevalence of the different symptoms was found during the 9-year period between ECRHS I and II.

**Table 3:** Prevalence (n (%)) of symptoms reported after food intake (n = 2,307).

	ECRHS I 1991–92	ECRHS II 2000–01	p value
Rash or itchy skin	101 (4.4)	112 (4.9)	0.39
Diarrhoea or vomiting	40 (1.7)	47 (2.0)	0.26
Runny or stuffy nose	42 (1.8)	48 (2.1)	0.42
Severe headache	18 (0.8)	25 (1.1)	0.21
Breathlessness	54 (2.3)	56 (2.4)	0.87
Other	131 (5.7)	149 (6.5)	0.047

### Natural history of prevalence of IgE sensitisation to food and aeroallergens

**Figure 2:**
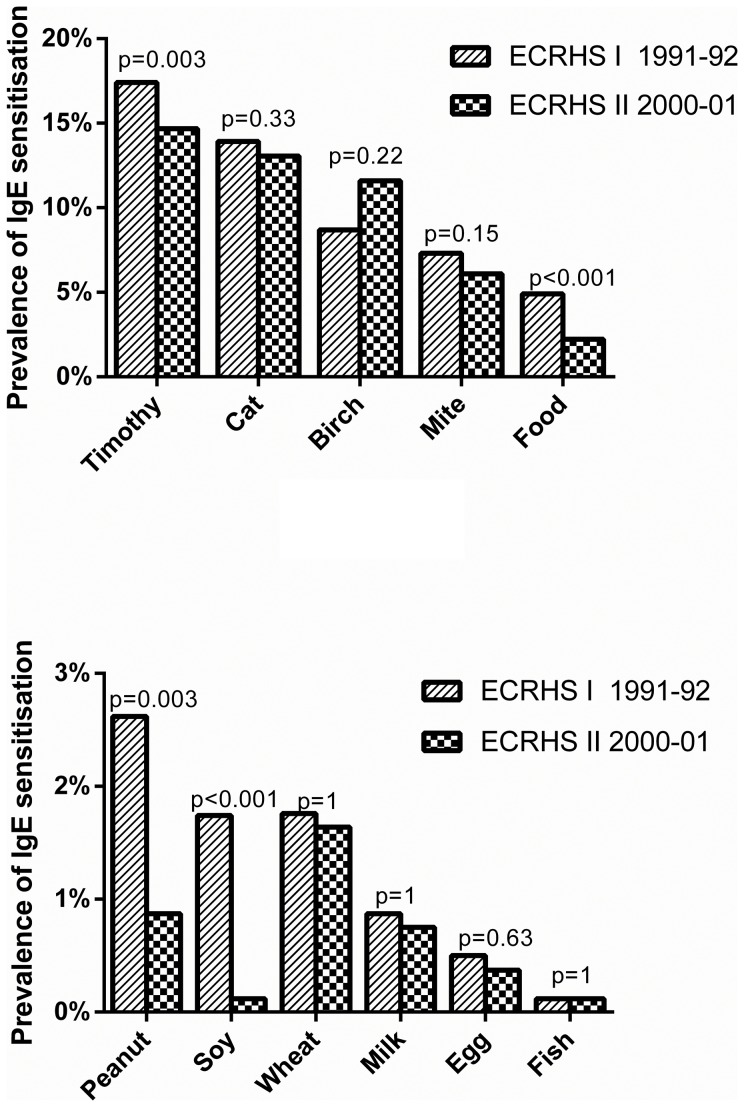
Prevalence (number of individuals of the study population who have IgE for respective allergen ≥0.35 KU/L) of sensitisation to food (n = 807) and aeroallergens (n = 804) (upper panel) and prevalence (number of individuals of the study population who have IgE for respective allergen ≥0.35 KU/L) of IgE sensitisation to individual food allergens (lower panel) in ECRHS I and II. Birch pollen was measured only in Uppsala (n = 138).

Prevalence of IgE sensitisation to at least one food allergen decreased by 56% during the study (Figure 2A). Prevalence of peanut IgE-sensitisation was the highest among food allergens and decreased by 67%. The prevalence of soy IgE sensitisation decreased significantly between ECRHS I and II, whereas similar prevalence of IgE sensitisation to wheat, milk, egg and fish was found at both time points (Figure 2B). No significant change of prevalence of cat, birch and mite IgE sensitisation was found, whereas prevalence of timothy grass IgE sensitisation decreased by 15% (from 17% to 15%) (Figure 2A).

The prevalence of IgE sensitisation to food allergens decreased from ECRHS I to ECRHS II by 63% (from 6% to 2%) (p<0.001) in subjects younger than 31 years and by 44% (from 4% to 2%) (p<0.001) in subjects older than 31 years (median age of cohort in ECRHS I).

### Changes over time of levels of IgE against food- and aeroallergens and total IgE

**Figure 3:**
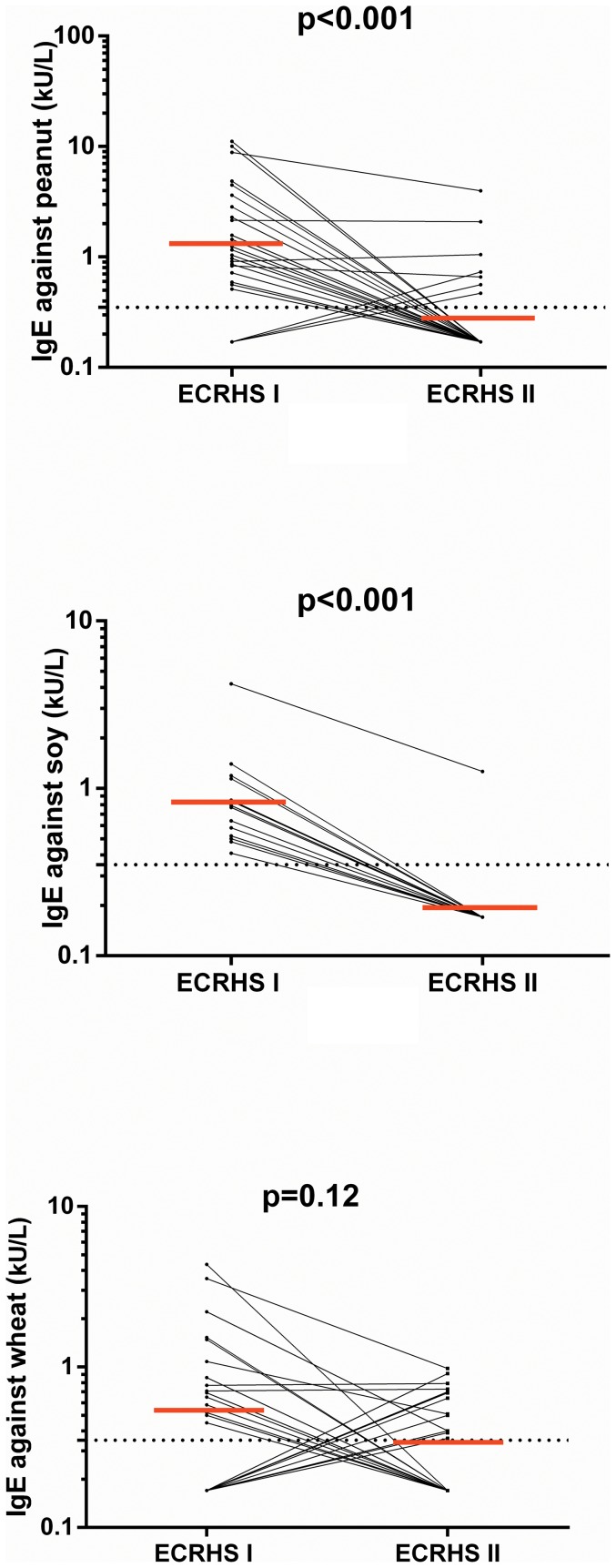
Change in levels of specific IgE against peanut (n = 24), soy (n = 14) and wheat (n = 22) between baseline and follow-up in subjects sensitised either at baseline or at follow-up. (The dotted line corresponds to the detection limit of 0.35 kU/L and the red lines correspond to the geometrical mean of levels of specific IgE against each respective allergen).

Significant decreases of IgE antibodies against peanut (p<0.001, Figure 3a) and soy (p<0.001 Figure 3b), but not against wheat (p = 0.12, Figure 3c), milk (p = 0.9), egg (p = 0.4) were found between ECRHS I and II among individuals who were IgE-sensitised to the respective allergen either at baseline or follow-up.

Similarly, among the aeroallergens, the levels of IgE antibodies against timothy grass decreased (p<0.001) whereas levels of IgE against cat (p = 0.18), birch (p = 0.43) and mite (p = 0.12) remained stable between ECRHS I and II among individuals who were IgE-sensitised to the respective allergen either at baseline or follow-up.

Total IgE increased between ECHRS I and II (17.7 (16.0, 19.7) vs. 25.7 (23.4, 28.2) kU/L (geometric mean) (p<0.001)).

### Incidence of food hypersensitivity

**Figure 4:**
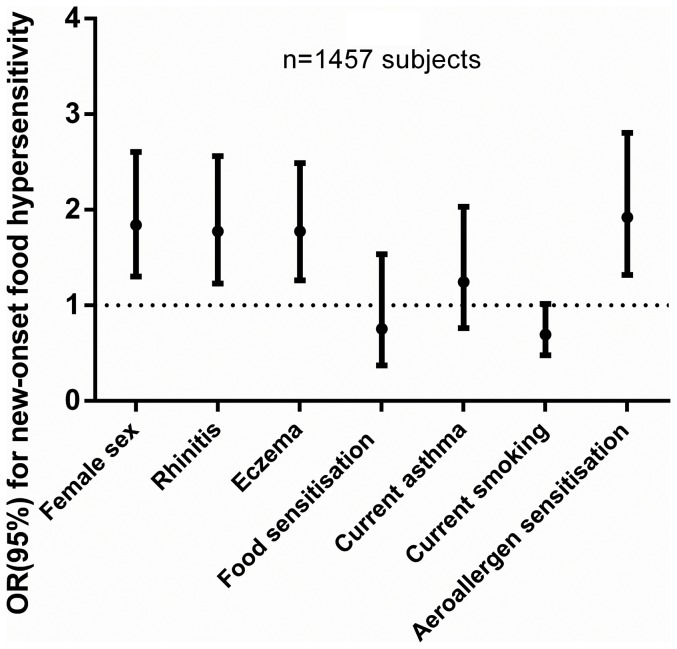
Risk factors (Odds Ratios (95%CI)) for new onset food hypersensitivity. Odds ratios are adjusted for the variables in the figure and age, body mass index, current smoking, sample and total IgE.

The incidence of food hypersensitivity was 11.7% during this 9-year study. The incidence in the symptomatic sample was higher than the random sample (20.6% vs. 9.9%, p<0.001). Female sex, rhinitis, eczema, asthma and presence of IgE sensitisation to aeroallergens were associated with new onset food hypersensitivity (Table 4) and these associations were consistent in a multiple logistic regression model (Figure 4).

**Table 4:** Population characteristics in different population strata, subdivided according to presence/absence of food hypersensitivity at ECRHS I and II.

	No food hypersensitivity (n = 1601)	New-onset food hypersensitivity (n = 211)	p value*
Age (years)	33.8±7.3	33.4±7.3	0.35
Female sex	48.8	61.6	<0.001
BMI (kg/m^2^)	23.7±3.3	23.5±3.4	0.43
Current smoker	31.6	26.5	0.133
Rhinitis	23.0	43.1	<0.001
Eczema	46.6	62.6	<0.001
Asthma	8.8	16.1	0.001
Total IgE (kU/L)	19.0 (18–20)	23.0 (18–29)	0.09
IgE sensitisation to food allergen	5.0	6.8	0.32
IgE sensitisation to aeroallergens	26.5	46	<0.001

Results presented as either prevalence (%), mean±SD or geometric mean (95% CI).

p-value for comparison new-onset food hypersensitivity vs. no food hypersensitivity

### Incidence and persistence of IgE sensitisation to food allergens

New onset IgE food sensitisation (n = 8) was not associated with age, gender, BMI, current rhinitis, current asthma, current smoking and IgE sensitisation to aeroallergens (data not shown). Persistent IgE food sensitisation was associated with higher total IgE levels at baseline (Table 5) and this association remained significant also after adjusting for age, gender, BMI, current rhinitis, current asthma, current smoking and IgE sensitisation to aeroallergens (OR (95% CI) = 9.8 (1.6, 60.5) per unit of natural logarithm-transformed total IgE). No relation between persistence of IgE sensitisation to food allergens and specific IgE levels to food allergens or aeroallergens or prevalence of IgE sensitisation to aeroallergens at baseline was found (Table 5).

**Table 5:** Baseline characteristic of subjects with persistent IgE food sensitisation compared to those with remission.

	Persistent IgE food sensitisation (n = 10)	Remission of IgE food sensitisation (n = 30)	p value*
Female sex	60.0	43.0	0.36
Age (years)	29.7 ±1.9	30.3 ±0.8	0.74
BMI (kg/m^2^)	23.2 ±1	24 ±0.6	0.55
Smoking	20.0	17.0	0.81
Rhinitis	50.0	80.0	0.07
Asthma	10.0	23.0	0.36
Food hypersensitivity	44.4	40.0	0.81
Total IgE (kU/L)	370 (110–1242)	106 (72–155)	0.008
IgE sensitisation to aeroallergens	9.0 (90)	24.0 (80)	0.47

Results presented as either prevalence (%), mean ± SD or geometric mean (95% CI).

p-value for comparison persistent IgE food sensitisation and remission of IgE food sensitisation

## Discussion

The main findings in the present investigation are that one in five adults reported food hypersensitivity symptoms after food intake and this proportion remained unchanged during this 9-year longitudinal study. The prevalence of IgE-sensitisation to food allergens decreased during that 9-year period whereas the prevalence of sensitisation to aeroallergens remained relatively unchanged.

To our knowledge this is the first study that analysed longitudinal changes of perceived self-reported food hypersensitivity in adults. The prevalence of food hypersensitivity reported here is in line with reports from previous cross-sectional studies [2]–[4], [28]. Our results showing an unchanged prevalence of food hypersensitivity over time are opposite to what is known about the natural history of food hypersensitivity in children, where major remission of symptoms has been reported [29]–[31].

The two most common reported symptoms of food hypersensitivity were skin reactions and gastrointestinal reactions. Reactions occurring at the skin or mucosa were also the most prevalent in a previous study [4]. Fruits, nuts and vegetables were the three most common causes of food hypersensitivity in our population, with a similar prevalence at baseline and follow-up. A previous study [4] showed the same major causes of adverse reactions to foods.

The risk factors for new onset food hypersensitivity in the present study were female sex, rhinitis, eczema, asthma and presence of IgE sensitisation to aeroallergens. Thus, incidence of food hypersensitivity seems to be related to the atopic trait of an individual. We are not aware of any published longitudinal study to compare our results with. However, there are cross-sectional studies showing that food hypersensitivity in adults is often associated with concomitant sensitisation to aeroallergens [4], IgE sensitisation to food allergens [32] and other manifestations of atopy, especially rhinitis [4].

A novel finding of the study was the marked reduction of prevalence of IgE sensitisation to food allergens over time in adults. The natural history of prevalence of IgE sensitisation to food allergens has previously been studied only in children [33] where sensitisation rates to food allergens remained stable during the first 6 years of life (10%). The majority of children outgrow their allergies to milk, soy, egg, and wheat, and some to peanut also [34]. The decrease in the prevalence of IgE sensitisation to food allergens does not appear to be related to the initial age of the subjects, which tends to preclude the possibility that the effect is related to a specific birth cohort. Reductions of similar size could be observed in both younger and older subjects. This probably signals that the decreasing prevalence is somehow related to aging. Previous longitudinal studies [12], [14], [16], [17] on the prevalence of IgE sensitisation to aeroallergens in relation to age report inconsistent results.

In the present study the prevalence of sensitisation to aeroallergens decreased to a much lesser extent than sensitisation to food allergens. However, the levels of IgE antibodies decreased during the 9-year follow-up period for both food and aeroallergens, whereas the levels of total IgE increased. Most of the longitudinal studies have analysed changes in the prevalence of IgE sensitisation without reporting changes in the degree of IgE sensitisation. A decrease of IgE titres to mite in a long-term follow-up of subjects with house-dust allergic asthma has been reported [35]. Karakaya and Kalyoncu [18] reported that the wheal diameter in response to skin prick test decreased under a two-year period for two of the studied allergens, timothy grass and mite. The reason why specific IgE decreased and total IgE increased in the present study is unclear. However our finding of increase total IgE is consistent with an increase of total IgE in all ECRHS centres [36] even if other longitudinal studies reported stable levels over time [17], [37]. The total IgE levels reflect more than the sum of specific IgE towards a broad panel of allergens [38]. Moreover the fraction of total IgE not-related to specific sensitisations was not related to the specific IgE fraction of total IgE [38]. The factors that control the fraction of total IgE that are not related to specific IgE sensitisation are not fully understood.

Subjects with higher total IgE levels at baseline were more likely to have persistent sensitisation to food allergens in our study. Increased levels of total IgE have been reported to predict the development of IgE sensitisation to aeroallergens and asthma symptoms in children [39]. As far as we know, no study has analysed the relation between baseline total IgE levels and persistence of IgE sensitisation to food allergens, whereas increased total IgE at baseline related [37] to persistence of aeroallergen IgE sensitisation in previous longitudinal studies.

The strength of this study is the population setting and the long follow-up period. This is the first study to our knowledge assessing food hypersensitivity and IgE levels against food allergens in a longitudinal setting. A weakness of the present study is that we were not able to assess the persistence/remission of food hypersensitivity as the questions refer to ever having food hypersensitivity. A limitation is the fairly limited number of participants for whom we had data from repeated measurements of IgE against food allergens. Another limitation is that several of the most common food allergens in adulthood, such as hazelnut, shrimp, peach and apple [27], were not analysed at both time points. The different freezing times before analysis of IgE for samples from ECRHS I and II and that measurements were performed in different laboratories (Uppsala for ECRHS I and Manchester for ECRHS II) does not appear to influence the measured IgE levels [40], [41]. Cross reactivity with IgE antibodies against birch or timothy grass can influence the measured IgE sensitisation to food allergens, and the lower prevalence of IgE sensitisation to food allergens at follow-up could have been due to a reduced prevalence of IgE sensitisation to birch at the follow-up. However, the prevalence of IgE sensitisation to birch did not decrease in the present study while the prevalence of IgE sensitisation to timothy grass decreased to a much lesser degree than IgE sensitisation to food allergens.

In conclusion, the prevalence of food hypersensitivity remained unchanged while IgE sensitisation to food allergens decreased in this 9 year follow up study performed in adults. The prevalence of IgE sensitisation towards food allergens decreased to a larger extent than the prevalence and persistence of IgE sensitisation to aeroallergens. The biological explanation for the high prevalence of food hypersensitivity should be further investigated.
